# Reduced exposure of imatinib after coadministration with acetaminophen in mice

**DOI:** 10.4103/0253-7613.56071

**Published:** 2009-08

**Authors:** Inthisham Nassar, Thanikachalam Pasupati, John Paul Judson, Ignacio Segarra

**Affiliations:** Departments of Pathology, International Medical University; No. 126, Jalan 19/155B, Bukit Jalil-57000 Kuala Lumpur, Malaysia; 1 Department of Human Biology, International Medical University; No. 126, Jalan 19/155B, Bukit Jalil-57000 Kuala Lumpur, Malaysia; 2 Department of Pharmaceutical Technology, International Medical University; No. 126, Jalan 19/155B, Bukit Jalil-57000 Kuala Lumpur, Malaysia

**Keywords:** Acetaminophen, chronic myeloid leukemia, drug–drug interaction, gastrointestinal stromal tumor, imatinib, pharmacokinetics

## Abstract

**Purpose:**

Imatinib is an efficacious drug against chronic myeloid leukemia (CML) and gastrointestinal stromal tumor (GIST) due to selective inhibition of c-KIT and BCR-ABL kinases. It presents almost complete bioavailability, is eliminated via P450-mediated metabolism and is well tolerated. However, a few severe drug-drug interactions have been reported in cancer patients taking acetaminophen.

**Materials and Methods:**

Male ICR mice were given 100 mg/kg single dose of imatinib orally or imatinib 100 mg/kg (orally) coadministered with acetaminophen intraperitoneally (700 mg/kg). Mice were euthanized at predetermined time points, blood samples collected, and imatinib plasma concentration measured by HPLC.

**Results:**

Imatinib AUC_0-12_ was 27.04 ± 0.38 mg·h/ml, *C*_max_ was 7.21 ± 0.99 mg/ml and elimination half-life was 2.3 hours. Acetaminophen affected the imatinib disposition profile: AUC_0-12_ and *C*_max_ decreased 56% and 59%, respectively and a longer half-life was observed (5.6 hours).

**Conclusions:**

The study shows a pharmacokinetic interaction between acetaminophen and imatinib which may render further human studies necessary if both drugs are administered concurrently to cancer patients.

## Introduction

The clinical response to drugs may be affected by the simultaneous administration of other drugs that modify the pharmacokinetics and the disposition profile of medications. Drug-drug interaction accounts for about 20-30% of all adverse reactions to drugs and are particularly important in the treatment and management of cancer patients to optimize dosing and manage toxicity associated with the cancer treatment.[1] Imatinib, is a rationally designed potent inhibitor of several protein kinases including BCR-ABL and cKIT. Currently, it is approved for the treatment of chronic myeloid leukemia (CML)[2] and gastrointestinal stromal tumors (GIST).[3] Imatinib has been shown to present a variety of drug-drug interactions including antibiotics,[4] immunosuppressant drugs,[5] cardiovascular agents[6] and antifungals[7] among others. The large majority of these interactions involve changes in clearance and bioavailability and have their origin in a common P450 biotransformation pathway which affects the pharmacokinetics and the disposition profile of imatinib such that may require dosing adjustment.[8]

The pharmacokinetic profile of imatinib in humans shows extensive absorption with almost complete bioavailability[9] although large intersubject systemic exposure (AUC) variability has been observed.[10] It is highly bound to plasma proteins (95%) mostly to α-1-acid glycoprotein and to a lesser extent to albumin, as well as to erythrocytes.[11] Imatinib is eliminated via hepatic metabolism to form the main metabolite *N*-demethylated piperazine which shows activity similar to the parent compound. Metabolism is largely mediated by CYP3A4 and CYP3A5 with minor contributions of other P450 isoforms, CYP1A2, CYP2D6, CYP2C9, and CYP2C19.[12] The elimination half-life was 18 hours and 40 hours for imatinib and its metabolite, respectively. Excretion eventually occurs through the bile into the intestine and only 5% is recovered unchanged in urine.[12]

Imatinib is well tolerated and presents a good safety profile at therapeutic doses (400 mg or 600 mg) with side effects that are reversible and dose dependent.[8,10] Hepatotoxicity is not a common adverse effect, but since imatinib's approval by the FDA in 2001 for the treatment of CML there have been a few reports of severe hepatic toxicity. In brief, imatinib increased levels of liver enzymes (AST and ALT) and bilirubin, caused hepatic dysfunction, liver damage including necrosis and hepatitis among others. Prolongation of prothrombin time was also observed. Patients experienced remission and recovery from toxicity upon discontinuation of imatinib treatment. However, in some patients there was extensive liver damage that led to death. Two of these cases refer to patients who were taking acetaminophen also. Histopathological analysis of the above-mentioned cases mainly showed hepatic necrosis and cytolytic hepatitis, but the pathogenic mechanisms of imatinib-induced hepatotoxicity were not established. It has been suggested that metabolite-induced toxicity can be due to a drug interaction between imatinib and other drugs that are metabolized via the cytochrome P450 pathway[13] as well as idiosyncratic reactions in susceptible patients.[14]

Acetaminophen is an analgesic and antipyretic agent whose main elimination is by glucuronidation and sulfation.[15] Biotransformation via cytochrome P450 by oxidative conversion catalyzed mainly by CYP2E1 and other enzymes CYP1A2, CYP2A6, CYP2D6, and CYP3A4[16] leads to the formation of *N*-acetyl-p-benzo-quinone imine (NAPQI) responsible for acetaminophen toxicity.[15] NAPQI irreversibly binds to intracellular glutathione forming a non-toxic glutathione conjugate which is further transformed to mercapturic acid and excreted in urine with the other conjugated metabolites. About 2% of acetaminophen is excreted unchanged.[17]

An interaction between imatinib and acetaminophen may affect the pharmacokinetic profile and hepatotoxicity of imatinib leading to severe damage.[18] The current study aims to assess the effect that coadministration of acetaminophen has on the pharmacokinetics and exposure of imatinib. Mice were chosen due to the extensive literature available for acetaminophen in these species and also because most of preclinical toxicology and cancer models have been established in mice. This will provide valuable information to assess the extent of the potential pharmacokinetic interaction between imatinib and acetaminophen *in vivo* and its effect on pharmacodynamic models.

## Materials and Methods

### Chemicals, reagents and dosing solutions

Imatinib was purchased from Cipla Ltd (Mumbai, India) and was stored protected from light at 4°C. Extraction and HPLC solvents such as methanol and acetonitrile (HPLC grade), glacial acetic acid, and triethylamine (analytical grade) were obtained from Fisher Scientific (UK). Orthophosphoric acid 85% (analytical grade) was purchased from Merck (Germany), sodium chloride (molecular biology grade) from Promega (USA) and EDTA-disodium salt from Merck.

Imatinib was dissolved in 0.9% saline solution and diluted ortho-phosphoric acid was added to obtain a pH of 4 and stored at 4°C protected from light. Acetaminophen suspension in 0.9% saline vehicle was vortex mixed, sonicated for 20 minutes and stired at 37°C until IP administration. All dosing solutions were prepared fresh for each administration.

### Animal studies and protocols

Male ICR mice (Institute of Medical Research, Kuala Lumpur, Malaysia) of similar age (range 12-14 weeks) and weight (30-35 g) were housed at the animal holding facility for acclimatization for at least 1 week and were provided water and food *ad libitum* in a 12-hour light cycle at 20 ± 2°C. The Institutional Animal Use and Ethics Committee reviewed and approved all the animal procedures prior to initiation of the study.

Animals were fasted overnight prior to dose administration. Four animals were dosed per time point (5, 10, 15, 30 minutes, 1, 2, 4, 6, 9 and 12 hours) as follows: Mice in group I were given 100 mg/kg imatinib orally (0.1 ml/ 20 g) using a 24G feeding needle with a 1 ml syringe (Terumo Corporation, Philippines). The mice in group II were given imatinib as described and acetaminophen intraperitoneally (700 mg/ kg) using a 25G needle. Oral administration was done first followed by the IP administration. The mice were euthanized at the predetermined time points by cervical dislocation, blood was collected by cardiac puncture, and transferred into microvials containing EDTA. The plasma was separated by centrifugation and transferred to microvials and stored at −30°C until analysis.

### Sample preparation and HPLC analysis

On the day of analysis, plasma samples were allowed to thaw in ice. A 100 μL of plasma sample aliquot was added with 100 μL of methanol; vortex mixed for 3 minutes and centrifuged (15,000 rpm, 10 minutes, 4°C). Then the supernatant was transferred into a HPLC vial and kept on ice until HPLC analysis in a Perkin Elmer system (PE Series 200 pump and UV/VIS detector with PE series 600 LINK chromatography interface and PE NCI 900 network interface) with TotalChrom software for data acquisition and integration. A 25 μL aliquot was injected into an Inertsil^®^ CN-3 column (150 × 4.6 mm, 5 μm particle size) and eluted with a mixture of 35% methanol and 1% triethylamine, pH 4.8 and flow rate of 1 ml/min, a modified method from previous reports.[19] Imatinib was detected at 268 nm, the retention time was 7.5 minutes and plasma imatinib concentration calculated using an external calibration curve prepared daily.

**Figure 1:**
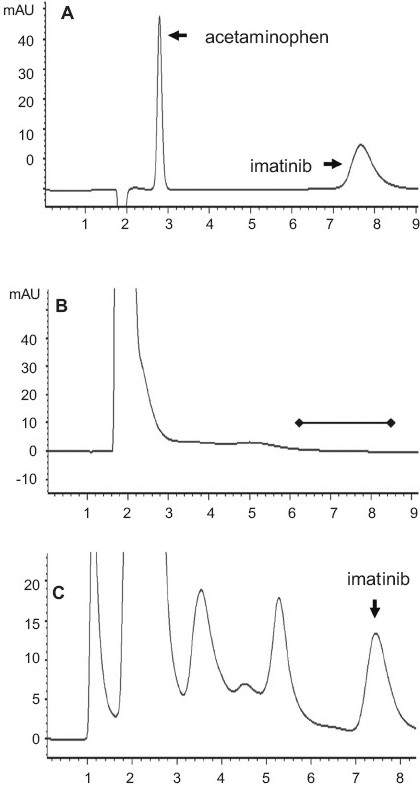
HPLC analysis of imatinib: (a) Chromatogram showing the absence of interference between acetaminophen and imatinib; (b) Chromatogram of a blank plasma sample showing the lack of matrix interference; (c) Chromatogram of a plasma sample at 40 min after coadministration of acetaminophen (700 mg/kg, IP) and imatinib (100 mg/kg, PO). The additional peaks at 3.6 and 5.5 min are likely to be imatinib metabolites.

The method was adapted from the literature and previously validated in our laboratory.[19] Linearity was evaluated within the range 0.01-80 μg/ml (8 concentration levels) but the assay performed linearly between 0.1 and 50 μg/ml (5 concentration levels) with r^2^ >0.999. Based on the percentage deviation from the nominal value, the sensitivity or lower limit of quantification (LLOQ) was established at 0.1 μg/ml (<20%) although imatinib was detected at 0.05 μg/ml. For the pharmacokinetic data analysis, plasma samples with concentration below 0.1 μg/ml were treated as zero. The assay was specific and no plasma matrix of acetaminophen interference was observed [Figure 1]. The assay was performed with less than 15% intraday and interday variabilities. Precision and accuracy at each concentration level of the calibration curve were also within the generally accepted value of 15%. Finally, imatinib recovery from the plasma matrix at two concentrations, 1.82 and 36.5 μg/ml, was 97.77% and 96.59%, respectively with CV lesser than 10%.

### Data analysis

Pharmacokinetic data analysis was done using non-compartmental approaches. The maximum concentration (C_max_) and time to maximum concentration (T_max_) were determined directly from the disposition profile graph. The area under the curve from zero to the last experimental concentration (AUC_0→t last_) was calculated using the log-trapezoidal rule. The extrapolated area was calculated as the last concentration divided by the elimination rate constant, C_last_/k_el_. The total imatinib exposure (AUC_0→∞_) was obtained adding both areas. The elimination rate constant, k_el_ was obtained from the log-linear regression of the terminal slope and half-life, t_1/2_ was ln2/k_el_. The mean residence time (MRT) was obtained dividing the AUMC (area under the first moment of the curve) by AUC and apparent clearance (Cl/F) was calculated as D/AUC_0→∞_ where D represents the dose. Then, the apparent volume of distribution at the steady-state V_SS_/F was calculated as MRT × Cl/F.

Differences in exposure (AUC_0→t last_) were analyzed using the *z*-test proposed by Bailer[20] for comparison of AUC for destructive sampling, when each experimental animal contributes with one sample.[21]

## Results

**Figure 2:**
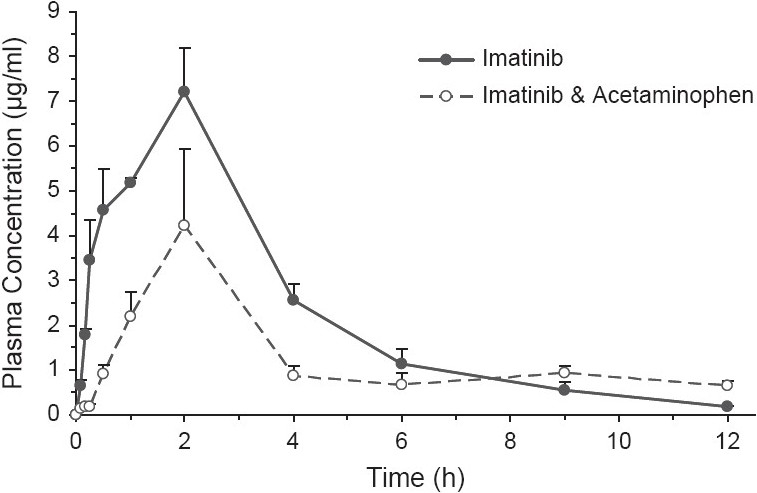
Plasma concentration-time curve profile of imatinib after oral administration to mice

The pharmacokinetic profile of imatinib in plasma after oral administration is shown in Figure 2 and the pharmacokinetic parameters calculated using non-compartmental techniques are listed in Table 1. Mice treated with 100 mg/kg imatinib PO showed a rapid increase in imatinib plasma concentration to reach the C_max_ (7.21 ± 0.99 μg/ml) at 2 hours (T_max_). Following the T_max_, the imatinib plasma concentration declined gradually with an elimination half-life of 2.3 hours. The total imatinib exposure or AUC_0-∞_ was determined to be 27.61 μg h/ ml, Cl/F was calculated to be 3.7 l/h/kg, and the MRT was 3.3 hours.

**Table 1:** Model-independent pharmacokinetic parameters of imatinib in mice after administration of 100 mg/kg dose orally (control) or with coadministration of 700 mg/kg dose of acetaminophen intraperitoneally

*Parameter*	*Imatinib*	*Imatinib and acetaminophen*
C_max_ (μg/ml)	7.21 ± 0.99	4.24 ± 1.69
T_max_ (h)	2	2
k_el_ (h^−1^)	0.320	0.124*
T_1/2_ (h)	2.3	5.6*
AUC_0→12_ (μg·h/ml)	27.04 ± 0.38+	15.66 ± 0.48+
AUC_0→∞_ (μg·h/ml)	27.61	20.91*
MRT_0→12_ (h)	3.03	4.40
MRT_0→12_ (h)	3.3	8.3
Cl/F (l/h/kg)	3.70	6.39
V_SS_/F (l/kg)	12.1	53.2

* Elimination rate constant estimated only from the last two time points. See the text for further explanation.

+ Standard error calculated according to the Bailer method[20]

Simultaneous administration of acetaminophen at 700 mg/kg IP affected the plasma pharmacokinetic profile of imatinib. Although the T_max_ was not changed, the C_max_ was lower, 4.24 ± 1.69 with *P* < 0.05 of significant level (*t*-test). The total AUC in group II could not be accurately determined due to the lack of accuracy in the calculation of the terminal slope and the elimination rate constant. Thus, to evaluate the effect of acetaminophen of imatinib exposure, the AUC from zero to the last time point with measured concentration (AUC_0-t last_) in each group was calculated. After IP administration of acetaminophen, the AUC_0-12_ was 15.66 ± 0.48 μg·h/ml versus 27.04 ± 0.38 μg·h/ ml in the group that received only imatinib with *P* < 0.01 after *z*-test analysis as proposed by Bailer.[20] The MRT_0-12_ was also greater when acetaminophen was administered concurrently (4.4 hours) than alone (3.03 hours) as well as Cl/F (calculated using AUC_0-12_) that was 6.39 l/h/kg for the study group versus 3.70 l/h/kg for the control group. Finally, V_SS_/F was also affected and increase from 12.1 l/kg (control group) to 53.2 l/kg in the study group.

## Discussion

After oral administration of 100 mg/kg of imatinib, the plasma concentration raise very fast during the first 30 minutes which suggests a fast absorption rate. Imatinib plasma concentration increase seems to slow down before reaching the C_max_ at 40 minutes which is followed by a gradual decline of the plasma concentration with a half-life of 2.3 hours. This half-life is slightly longer than that previously reported of 1.3 hours.[2] However, this discrepancy could be attributed to differences in dose between both studies, or to the fact that their study may not portray a full pharmacokinetic profile, as sampling was done only up to 2 hours after imatinib administration.

The concentration-time profiles of imatinib levels in plasma clearly show the effect of acetaminophen on imatinib pharmacokinetics after 700 mg/kg acetaminophen co-administration [Figure 2]. The selection of the acetaminophen dose was done based on two criteria: First, the clinical scenarios where cancer patients who are receiving daily imatinib treatment may take a high dose of acetaminophen, which can lead to overdose, for pain relief. Second, the desired dose may be high but should not lead to hepatic damage since imatinib is mostly eliminated via metabolism. A study in rats, that evaluated the hepatic functionality based on alteration of the liver enzymes in serum after acetaminophen IP administration (range 250 mg/kg-1000 mg/kg) only showed liver enzyme changes in plasma at 1000 mg/kg. Taking into account this information, a 700 mg/kg dose of acetaminophen was selected to mimic a high dose but avoiding significant liver damage. Finally, the route of administration would be IP to minimize the possible chemical interaction between acetaminophen and imatinib.

The comparison of both plasma disposition profiles shows a 56% decrease in AUC_0-12_ which is largely significant (*P* <0.01) based on the methodology proposed by Bailer for comparison of AUC when the pharmacokinetic profile is develop using destructive sampling.[20] In addition, there was also a 59% reduction of imatinib plasma C_max_ when imatinib is co-administered with acetaminophen which suggests a decrease in the dose absorbed, assuming that imatinib obeys linear pharmacokinetics at this dose. In addition, there seems to be a slight delay in the absorption at the initial stages up to 15 minutes where some imatinib plasma concentrations were below the LOQ. The delay is also in parallel to a slight increase in MRT, which could be due to some unspecific effect of the IP administration. Moreover, similar effect of acetaminophen on drug exposure has been also documented in veterinary medicine.[22] In a pharmacokinetic study of doxycycline in swine to optimize therapy against porcine respiratory disease, it was observed that co-administration of acetaminophen led to a reduction of doxycycline plasma AUC and C_max_. However, the authors did not address the mechanism involved other than suggesting a lesser feed intake.

Changes in imatinib exposure (AUC) could be due to a change in clearance (Cl), bio-availability (F) or both and may be explained taking into account the relationship between them as shown in the following equation where D is the dose given: $AUC=\frac{F\times D}{Cl}$

Thus, changes in imatinib clearance and bioavailability caused by acetaminophen could affect the disposition and exposure of imatinib. The decrease in bioavailability may be explained by a possible increased presence or functionality of p-glycoprotein and other efflux transporters due to the co-administration of acetaminophen. Increased expression of mRNA and hepatocellular efflux transport proteins levels including Mrp4, Mrp5, p-glycoprotein, and BCRP was observed in mice treated with acetaminophen that had developed hepatotoxicity.[23] Similarly, increased expression of Mrp1, Mrp3, and Mrp4 after administration of acetaminophen was also observed in mice[24] and in rats.[25] Thus, based on these studies, and given the fact that imatinib has been proven to be a substrate of p-glycoprotein,[26] MDR1,[27] and other transporters,[28] it may be suggested that the decreased exposure could be related to an increase of transporter proteins (including p-glycoprotein) triggered by acetaminophen or an increase of their functionality leading to higher bile excretion which in combination with lower absorption would result in lower systemic exposure. In addition, a mechanism for which imatinib AUC may be reduced is an increase in clearance: It has been also shown that p-glycoprotein increases imatinib clearance in mice.[29] An increase of cytochrome P450 enzymes CYP2E1 and CYP3A levels was observed in rats after administration of 500 mg/kg dose of acetaminophen.[30] In our study, carried out in mice instead of rats, the dose used was higher, which would suggest some degree of CYP3A enzymes increase. Since imatinib is metabolized into an active metabolite, CGP74588, mainly by CYP3A4 and CYP3A5 (CYP2D6 and CYP2C9 are minor contributors),[8,12] it may undergo more extensive metabolism in the presence of acetaminophen. However, this last mechanism seems less likely as it would require longer exposure to induce metabolism. Alternatively, the presence of acetaminophen may increase the unbound, free fraction in plasma making more imatinib available for metabolism. The combination of these two mechanisms, a reduction in bioavailability and an increase of metabolism, could contribute to an overall reduced systemic exposure of imatinib. Unfortunately, our HPLC assay did not allow for the quantification of imatinib metabolites, which would help to clarify the contribution of each one of this suggested mechanism. This bioanalytical limitation prevents a solid conclusion toward the separate contribution of p-glycoprotein and CYP3A4 to decrease imatinib AUC.

Finally, the large increase observed for Cl/F and V_SS_/F may also reflect the effects of acetaminophen in the overall bioavailability: Cl/F shows a two-fold increase which is parallel to the ∼50% decrease in AUC. However, V_SS_/F has a larger increase (∼ 4-fold) suggesting additional effects affecting the V_SS_, probably on imatinib distribution, due to the coadministration of acetaminophen. This increase in V_SS_/F also brings another important aspect to the coadministration of acetaminophen to alleviate pain in cancer patients undergoing imatinib treatment. Besides the change in AUC, there may be an increase in the tissue concentration that leads to an increase in V_SS_/F. This may have serious clinical implications regarding imatinib-based renal toxicity[31] and hepatotoxicity.[14] Severe hepatotoxicity that includes several fatal cases has been reported in cancer patients taking imatinib and acetaminophen for pain relief.[18,32] Thus, the co-administration of acetaminophen with imatinib should probably include liver and renal function monitoring to ensure that toxicity is not developed.

## Conclusions

This study shows a pharmacokinetic interaction between acetaminophen and imatinib when both drugs are administered concurrently. These findings are relevant for cancer therapeutics since acetaminophen is commonly used in pain management in cancer patients undergoing imatinib treatment. This may require some degree of dosing adjustment in patients who have been prescribed acetaminophen or who are prompt to take it to alleviate cancer-associated pain.

This research also shows the need to perform further studies in oncology patients to validate the findings from the preclinical model. Furthermore, the elucidation of the mechanism of the interaction would assist to optimize the therapeutic plan for cancer patients.
